# Unraveling the Position Effect of Spiroxanthene-Based n-Type Hosts for High-Performance TADF–OLEDs

**DOI:** 10.3390/nano13182517

**Published:** 2023-09-08

**Authors:** Qinglin Liu, Yun Deng, Baoyi Ren, Xia Lan, Yuehong Zhang, Runda Guo, Chensheng Li, Gang Xiong, Yaguang Sun, Zujin Zhao

**Affiliations:** 1 College of Science, Key Laboratory of Inorganic Molecule-Based Chemistry of Liaoning Province, Shenyang University of Chemical Technology, Shenyang 110142, China; lql1347585318@163.com (Q.L.); lanxia0928@163.com (X.L.); zyhrrmhs@126.com (Y.Z.); lichensheng_hi@163.com (C.L.); xg@syuct.edu.cn (G.X.); sunyaguang@syuct.edu.cn (Y.S.); 2 Frontiers Science Center for Flexible Electronics, Xi’an Institute of Flexible Electronics and Xi’an Institute of Biomedical Materials & Engineering, Northwestern Polytechnical University, Xi’an 710072, China; dengyun1981@163.com; 3 Wuhan National Laboratory for Optoelectronics, Huazhong University of Science and Technology, Wuhan 430074, China; 4 State Key Laboratory of Luminescent Materials and Devices, Key Laboratory of Luminescence from Molecular Aggregates of Guangdong Province, South China University of Technology, Guangzhou 510640, China; mszjzhao@scut.edu.cn

**Keywords:** TADF–OLED, host, spiro[fluorene-9,9′-xanthene], position effect

## Abstract

For developing high-performance organic light-emitting diodes (OLEDs) with thermally activated delayed fluorescent (TADF) emitters, the diphenyltriazine (TRZ) unit was introduced onto the 2′- and 3′-positions of xanthene moiety of spiro[fluorene-9,9′-xanthene] (SFX) to construct n-type host molecules, namely 2′-TRZSFX and 3′-TRZSFX. The outward extension of the TRZ unit, induced by the *meta*-linkage, resulted in a higher planarity between the TRZ unit and xanthene moiety in the corresponding 3′-TRZSFX. Additionally, this extension led to a perched *T*_1_ level, as well as a lower unoccupied molecular orbital (LUMO) level when compared with 2′-TRZSFX. Meanwhile, the 3′-TRZSFX molecules in the crystalline state presented coherent packing along with the interaction between TRZ units; the similar packing motif was spaced apart from xanthene moieties in the 2′-TRZSFX crystal. These endowed 3′-TRZSFX superior electron transport capacity in single-carrier devices relative to the 2′-TRZSFX-based device. Hence, the 3′-TRZSFX-based TADF–OLED showed remarkable electroluminescent (EL) performance under the operating luminance from turn-on to ca. 1000 cd·m^−2^ with a maximum external quantum efficiency (*EQE*_max_) of 23.0%, thanks to its matched LUMO level with 4CzIPN emitter and better electron transport capacity. Interestingly, the 2′-TRZSFX-based device, with an *EQE*_max_ of 18.8%, possessed relatively low roll-off and higher efficiency when the operating luminance exceeded 1000 cd·m^−2^, which was attributed to the more balanced carrier transport under high operating voltage. These results were elucidated by the analysis of single-crystal structures and the measurements of single-carrier devices, combined with EL performance. The revealed position effect of the TRZ unit on xanthene moiety provides a more informed strategy to develop SFX-based hosts for highly efficient TADF–OLEDs.

## 1. Introduction

As the high technology for information display and solid lighting, organic light-emitting diodes (OLEDs) have drawn great attention across academia and industries [1]. For realizing 100% exciton utilization under an external applied electric field, currently, the phosphorescent metallic complexes and purely organic thermally activated delayed fluorescent (TADF) materials have been energetically explored as emitters in the emission layer (EML) of OLEDs [2,3,4]. Especially for TADF emitters that have minimized singlet-triplet energy gaps derived from the suitable separation of frontier molecular orbital (FMOs) via twisted donor-acceptor (D-A) or multi-resonance type structures, the third-generation purely organic emitters have been studied intensively to further boost the performance and lowering the cost of OLEDs [5,6,7]. Excitingly, the maximum external quantum efficiency (*EQE*_max_) of TADF–OLED reported by Yang and co-workers has come up to 40% [8].

Due to the charge transfer (CT) characteristics caused by FMO separation, the emission properties of TADF emitters under applied bias are sensitive to their embedding environment [9,10,11]. Hence, the host materials that mainly take on the duty of carrier transport and subsequent energy transfer are crucial for developing efficient and stable TADF–OLEDs. Initially, to avoid concentration quenching, the emitters are co-doped with host materials into the EMLs at relatively low concentrations typically, in which the host material with electric activity acts as a solid solvent to disperse the emitter. More importantly, the host materials will play unique roles through charge/energy transfer and, thus, stand up for the triplet-involved TADF emission. For a brilliant host material, the three essential features are desired: (i) appropriately high first triplet excited level (*T*_1_) for ensuring non-reverse energy transfer from hosts to light-emitting guests; (ii) excellent carrier injection/transport capacity with bipolar or even n-type characteristics for lowering operation voltage and enlarging exciton recombination region; and (iii) rigid and/or twisted configuration to control the interactions of host–host and host–guest for maintaining the abovementioned intrinsic properties of host molecules and enhancing the morphological stability of EMLs. To meet these desirable requirements of high-performance hosts, numerous efforts have been undertaken to ascertain the rational design principle on various skeletons [12,13].

According to the charge transport properties, the host materials can also be classified into three main categories: hole-transporting (p-type), electron-transporting (n-type), and bipolar. Relative to p-type hosts, the n-type and bipolar hosts show a much smaller difference between the hole and electron transport that would induce a wide emission zone and prolong the device’s lifetime by suppressing triplet–triplet annihilation [14]. In purely organic molecules, the carbon and hydrogen of predominant component elements are low electronegative, resulting in the hole mobility being at least one order higher than the electron mobility in most cases [15]. Endowing the host materials with bipolar or n-type characteristics hinges on the introduction of electron-withdrawing groups or units, such as the cyano group [16], diphenylphosphine oxide (DPO) [17], diphenyltriazine (TRZ) [18], etc. For instance, the DPO unit with the insulating and steric effects has performed important roles in elevating the *T*_1_ level and improving the bipolar property, and then the DPEPO has become the most popular host for TADF–OLEDs [19,20,21]. It is a wise design that inarching the high-performance, electric-activity unit on a rigid three-dimensional (3D) scaffold to regulate the performance of corresponding host materials. When introducing the DPO unit onto the 4′-position of spiro[fluorene-9,9′-xanthene] (SFX), a universal host named SFXSPO was successfully obtained for high-efficiency full-color and white TADF–OLEDs [22]. This result revealed the significance of twisted 3D molecular configuration for host materials. Encouraged by the excellent photoelectronic properties and thermal stability of the TRZ unit, Adachi and co-workers designed the n-type hosts by functionalizing the spirobifluorene (SBF) with the TRZ unit and realized the efficient and stable TADF–OLEDs. For example, the host SF3-TRZ has been widely acknowledged and employed for fabricating TADF model devices [23,24,25,26].

**Scheme 1:**
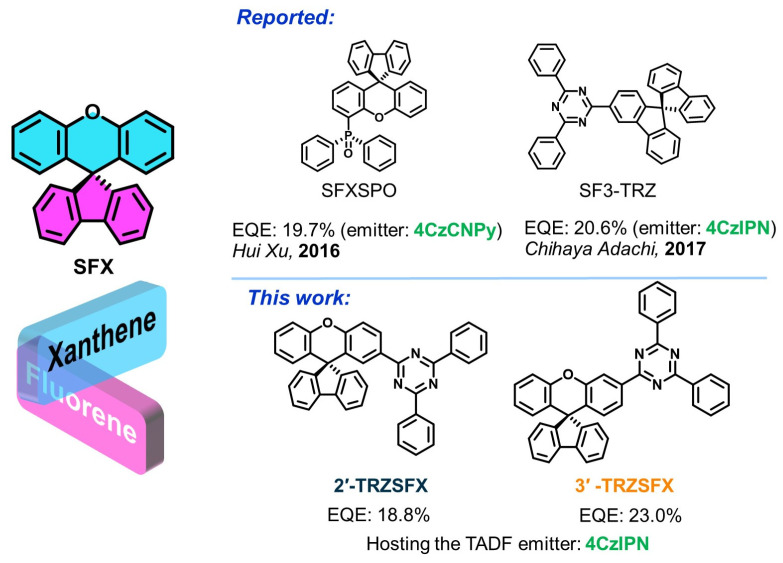
Reported n-Type hosts [22,23] and the molecular model in this work.

To maintain the advantage of n-type units in host molecules in a solid state, spiro polycyclic aromatic skeletons are ideal platforms by which the molecular packing can be adjusted, and the morphological stability of EML can be guaranteed. As far as spiro polycyclic aromatic compounds, the SFX is rising rapidly due to its lower synthetic cost and the matched or even superior performance relative to the classical SBF, as demonstrated by the phosphorescent emitters of *fac*-Ir(SFXpy)_3_ [27], host materials of SFXSPO [22] as well as hole-transport materials of HTM-FX′ [28]. For the binary spiro-structure of SFX, the xanthene moiety has more twisted conformation and higher *T*_1_ compared with the fluorene moiety [29,30]. In light of this, we designed the host materials, namely 2′-TRZSFX and 3′-TRZSFX (Scheme 1), by functionalizing the xanthene moiety of SFX using the certified n-type TRZ unit for TADF-OLEDs.

In this contribution, the difference between *para*-(2′-positon) and *meta*-(3′-positon) functionalization of *O*-atom of SFX with TRZ unit were comparatively studied. The two hosts both possessed impressive performance for TADF-OLEDs, surpassing the reported and wildly employed SF3-TRZ host. Interestingly, the pair of isomeric hosts presented subtle distinctions in the efficiency and roll-off in the electroluminescent (EL) process. Through the analysis of molecular structures, intrinsic optoelectronic properties and device performance, the relationship between the substituted position of the TRZ unit and the carrier transport property that is closely related to their EL manifestation was illustrated.

## 2. Materials and Methods

### 2.1. General Methods

The ^1^H/^13^C NMR spectra were obtained with Bruker Avance500 II spectrometers at ambient temperature by utilizing deuterated chloroform (CDCl_3_) as solvent and tetramethylsilane (TMS) as a standard. Mass spectra were obtained with a Waters Xevo G2-XS Tof. Single-crystal X-ray diffraction measurements of the crystals were performed with a Bruker Smart D8 Quest diffractometer equipped with a graphite monochromator. The determination of the unit cell parameters and the data collection were performed with Mo *K*_α_ radiation (λ = 0.71073 Å). UV-vis absorption spectra were measured using a UV-2550 Spectrophotometer. PL spectra were performed using an LS55 Spectrofluorophotometer. Cyclic voltammetry (CV) experiments were employed to evaluate the energy levels with a CH1760E Microcomputer-based Electrochemical Analyzer, using *n*-Bu_4_NPF_6_ (0.1 M) in dry dichloromethane as electrolyte solution and ferrocene as an internal reference. The experiments were carried out in a conventional three-electrode configuration, using a glass carbon disk as the working electrode, a saturated calomel electrode (SCE) as the reference electrode and a Pt wire as the counter electrode at a scan rate of 100 m Vs^−1^ under the protection of nitrogen. The 2′-TRZSFX and 3′-TRZSFX were dissolved in dichloromethane on the working electrode for measurements. The energy levels of the compounds were estimated by the equation: (assuming the energy level of ${FeCp}_{2}^{+/0}$ to be 4.8 eV in vacuum): *E*_HOMO_ = − [$E_{onset}^{ox}$ + 4.8] (eV); *E*_LUMO_ = − [$E_{onset}^{red}$ + 4.8] (eV). Thermogravimetric analyses (TGA) were determined using a METTL TOLEDO thermogravimetric analyzer under a heating rate of 10 °C min^−1^ and a nitrogen flow rate of 50 cm^3^ min^−1^.

### 2.2. OLED Fabrication

All purchased reagents and raw materials used in the synthesis process were used without further purification unless other stated. All reaction processes were carried out under the protection of a nitrogen atmosphere. The final compounds were further synthesized through a Suzuki cross-coupling reaction, as shown in the experimental section. The final compounds with little impurity were preliminarily purified using silica gel column chromatography, then repeatedly using recrystallization, and finally sublimation by gradient heating under vacuum conditions. For device fabrication, the used ITO-coated glass substrates (20 Ω square^−1^), MoO_3_, LiF, TAPC, TmPyPB and *m*CP were commercially available. The ITO substrates were dipped into acetone, isopropyl alcohol and deionized water orderly, and cleaned with an ultrasonic washer for 30 minutes. Before preparing devices, the ITO substrates were pretreated with oxygen plasma for 5 minutes. The devices were fabricated by evaporating organic materials, which were deposited in the vacuum of 2 × 10^−4^ Pa and monitored via a crystal oscillator system. The *J-V-L* property and EL spectra of the devices were measured by a Keithley 2400 source meter equipped with a PR655 spectrometer, and the emitting areas were 0.09 cm^2^, which was determined by the overlaps of two electrodes. All measurements were carried out at room temperature under ambient conditions.

## 3. Results and Discussion

### 3.1. Synthesis and Structure Characterization

**Figure 1:**
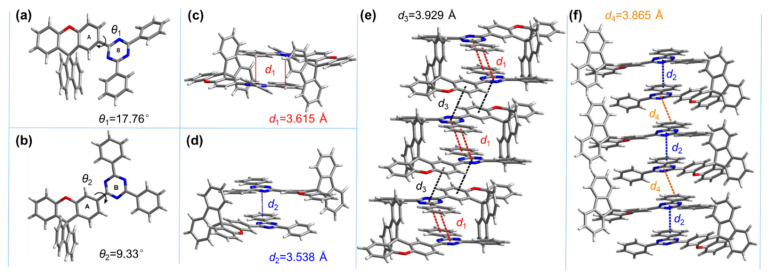
Molecular single crystal. (**a**) Twist angle of 2′-TRZSFX. (**b**) Twist angle of 3′-TRZSFX. (**c**) Dimer of 2′-TRZSFX. (**d**) Dimer of 3′-TRZSFX. (**e**) Stacking motif in 2′-TRZSFX crystal, *d*_1_ is the centroid–centroid distance in the dimer, and *d*_3_ is the distance between dimers. (**f**) Stacking motif in 3′-TRZSFX crystal, *d*_2_ is the centroid–centroid distance in the dimer, and *d*_4_ is the distance between dimers.

Based on our research interest in exploiting SFX for high-performance optoelectrical materials [27,31,32] and inspired by a current n-type host structure of SF3-TRZ for TADF-OLEDs [18,25,33], we employed a TRZ unit to functionalize the xanthene moiety of SFX. Except for endowing the hosts with n-type characteristics, there are two points to be considered for the modification. One is that the xanthene moiety possesses a dihedral angle of about 155° between its two phenyl rings [22]; hence, the nonplanar configuration would further assist the spiro-structure of SFX to weaken the intermolecular interactions of host–host and host–guest in the EML. The other one is that the insulating effect of the *O*-atom adopting sp^3^ hybridization would help the host molecules maintain high *T*_1_ energy and n-type characteristic, especially for 3′-TRZSFX with a TRZ unit at the *meta*-position of the *O*-atom. The synthesis routes of the two host materials 2′-TRZSFX and 3′-TRZSFX, were depicted in Scheme S1. The essential raw materials of 2′-BrSFX and 3′-BrSFX were prepared through a key Huang’s one-pot condensation. Significantly, the *meta*-bromo-substituted 3′-BrSFX which is unavailable via a classical electrophilic reaction of aromatic hydrocarbons was subtly synthesized by using a three-components strategy [34,35]. Subsequently, the Miyaura borylation was performed to get the corresponding boronic acid pinacol ester of SFXs, and then the TRZ unit was grafted on the xanthene moiety of SFX to assemble the target molecules through the Suzuki–Miyaura coupling. The molecular structures of both hosts were ascertained using ^1^H/^13^CNMR spectroscopy (see Supplementary Data), and single-crystal X-ray diffraction (Figure 1).

Figure 1 shows the molecular configurations, dimer interactions and the intermolecular packing motifs of the hosts in crystalline state. As expected, the bent xanthene planes of 2′-TRZSFX and 3′-TRZSFX had nearly the same dihedral angles of 155.9° and 156.8° between the two phenyls (Figure S1a,c), respectively. Hence, the planar fluorene tilted toward one side, resulting in the intersection angles of 80° and 81° (Figure S1b,d) between the fluorene and the xanthene for 2′-TRZSFX and 3′-TRZSFX, respectively. In 2′-TRZSFX molecule, the dihedral angle (*θ*_1_, Figure 1a) between triazine ring (A plane) and its linked benzene ring (B plane) of xanthene was 17.76°; however, this angle in 3′-TRZSFX molecule reduced to 9.33° (*θ*_2_, Figure 1b) because of the absent steric hindrance between the phenyl rings of TRZ unit and the fluorene moiety of SFX. In the dimer of 2′-TRZSFX (Figure 1c), the dual π···π interactions of adjacent TRZ units were observed through their benzene rings and triazine rings with the centroid–centroid distances of 3.615 Å (*d*_1_). For the dimer of 3′-TRZSFX, the TRZ units showed a more compact packing between a benzene ring and a triazine ring with the centroid–centroid distances of 3.538 Å (*d*_2_, Figure 1d), but the interaction was solely due to the relative dislocation between TRZ units with respect to the 2′-TRZSFX dimer. Figure 1e presents the packing motif of 2′-TRZSFX in a crystalline state. It was found that the packing between TRZ units in its dimer was spaced apart from the xanthene moiety with the centroid–centroid distance of 3.929 Å (*d*_3_) between the triazine ring and the benzene ring of xanthene. In the single crystal of 3′-TRZSFX, the coherent packing motif occurred throughout those n-type TRZ units (the distance between 3′-TRZSFX dimers, *d*_4_: 3.865 Å, Figure 1f), we speculate that much higher planarity (dihedral angle: 9.33°) between TRZ unit and xanthene moiety promoted the packing pattern [36]. The more consistent interactions between n-type TRZ units in 3′-TRZSFX crystal implied its superior electron transport capability than 2′-TRZSFX in EML.

The two hosts inherited the outstanding thermal stability of their building units of SFX and TRZ; the thermal decomposition temperatures (*T*_d_, 5% weight loss) measured from the thermogravimetric analysis were as high as 375 and 370 °C (Figure S2) for 2′-TRZSFX and 3′-TRZSFX, respectively. Although the measurements of differential scanning calorimetry did not give the *T*_g_ values, the high enough melting points (*T*_m_) of 298.8 and 295.5 °C (Figure S3) for 2′-TRZSFX and 3′-TRZSFX suggested good morphological stability of the hosts in solid film [37]. That is, the high enough *T*_d_ and *T*_m_ would guarantee the chemical and device stability of the host materials in the process of carrying out thermal evaporation and electro-excitation.

### 3.2. Theoretical Simulation and Electrochemistry

**Figure 2:**
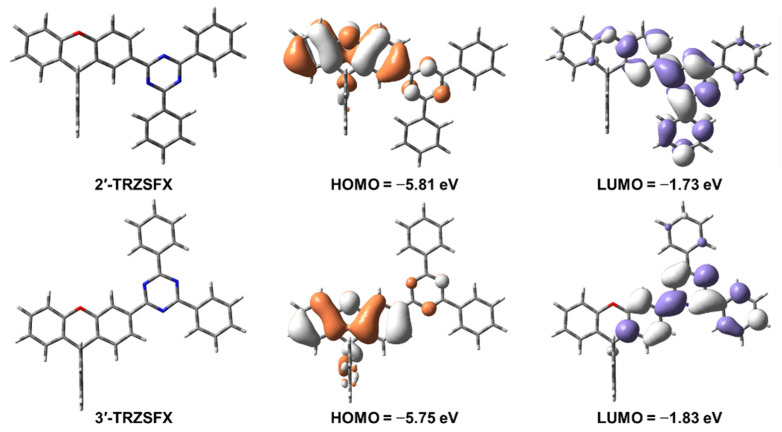
Distribution of HOMOs and LUMOs of 2′-TRZSFX and 3′-TRZSFX.

To evaluate the FMO distributions and energy levels of the host molecules, density functional theory (DFT, orbital distribution) and time-dependent DFT (TDDFT, energy level) calculations were performed using the B3LYP/6-31G (*d*, *p*) in the gas phase. As summarized in Figure 2, the HOMO of 2′-TRZSFX was mainly distributed on the xanthene moiety with part extension to the triazine ring, and the LUMO distribution basically occupied the TRZ unit and extended to the benzene ring and *O*-atom of xanthene moiety due to the *para*-linkage, indicating that the excited state of 2′-TRZSFX existed a degree of the mixture of a CT state and a localized state (LE). Relatively speaking, the *meta*-linked 3′-TRZSFX had decreased FMO overlap between TRZ unit and xanthene moiety; the difference in the distribution of FMOs may be partly an example of the so-called quantum interference [38]. Given the planar conformation of the D-A fragment in 3′-TRZSFX, the nature of the excited state remained to be estimated according to the photophysical test, which will be discussed later. The calculated HOMO/LUMO energy levels of the 2′-TRZSFX and 3′-TRZSFX were −5.81/−1.73 eV and −5.75/−1.83 eV, respectively. The determined LUMO and HOMO levels by cyclic voltammetry were −6.23 eV/−2.62 eV and −6.17 eV/−3.15 eV for 2′-TRZSFX and 3′-TRZSFX, respectively (Figure S4). The *meta*-linkage and enhanced planarity deepen the LUMO of 3′-TRZSFX. The HOMO levels of the n-type hosts were both deeper than general TADF emitters, which would promote the hole transport of emitting molecules and avoid the formation of high-energy excitons in the EL process [18]. In addition, the stabilized LUMO of 3′-TRZSFX would be conducive to electron injection and transport in EL devices.

### 3.3. Photophysical Properties

**Figure 3:**
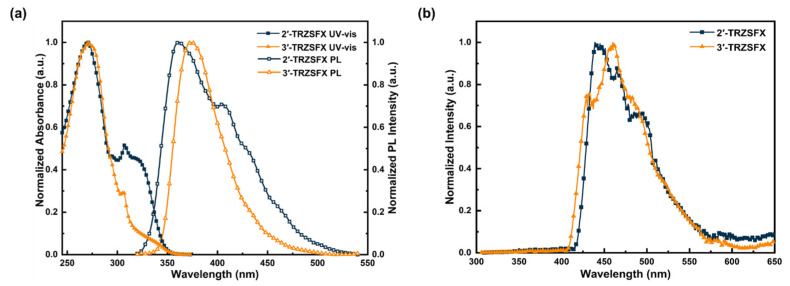
Molecular photophysical properties. (**a**) Normalized absorption and fluorescence spectrum of 2′-TRZSFX and 3′-TRZSFX (excitation: 310 nm) in dilute toluene (10^−5^ M). (**b**) Phosphorescent spectra of 2′-TRZSFX and 3′-TRZSFX in toluene at 77 K.

Figure 3a presented the ultraviolet–visible (UV-vis) absorption and photoluminescent (PL) spectra of 2′-TRZSFX and 3′-TRZSFX in toluene solutions (10^−5^ M) at 298 K. The two hosts had almost the same absorption locations. The absorption band from 250 to 315 nm was ascribed to the π→π* and the n→π* transitions of the fluorene moiety and the xanthene moiety of SFX, respectively. At the region of 320~355 nm, which was assigned to the CT transition between xanthene and TRZ, 2′-TRZSFX showed distinctly increased absorption relative to 3′-TRZSFX. This could be accounted for the strong CT characteristic of 2′-TRZSFX. Their features of absorption spectra were reflected in the corresponding PL profiles. For 2′-TRZSFX, the fluorescent (FL) spectra showed a strong emission located at 360 nm and a shoulder peak at 405 nm. The strong peak was assigned to the radiative transition of the LE state that involved of TRZ unit and coupling benzene ring, and the relatively weak shoulder peak was attributed to the CT emission from the xanthene donor to the TRZ acceptor, attributable to the *para*-linkage and twisted D-A structure. However, the 3′-TRZSFX presented a single emission peak at 375 nm, which was signed to the radiative transition of the LE excited state of the D-A conjugated segment, and the red shift was owing to its planar configuration relative to 2′-TRZSFX. The optical gap energies ($E_{g}^{\mathrm{opt}}$), deduced from the intersection point of the normalized UV-vis and FL spectra, were 3.68 eV for 2′-TRZSFX and 3.61 eV for 3′-TRZSFX, respectively.

Further, we measured the phosphorescent (PH) spectra of the hosts in toluene solution at 77 K, as depicted in Figure 3b. In the frozen matrix, the PH spectra clearly showed the vibrational structures with the phosphorescent onsets of 415 and 406 nm for 2′-TRZSFX and 3′-TRZSFX, respectively. It was worth noting that 3′-TRZSFX possessed a slight blue-shift emission with respect to 2′-TRZSFX, opposite of the tendency in FL spectra (Figure 3a). We speculated that the planar configuration gave rise to the intensified LE feature of 3′-TRZSFX, but the CT nature was more significant for 2′-TRZSFX in the PH emission. The singlet/triplet energies of 2′-TRZSFX and 3′-TRZSFX were determined to be 3.44/2.82 eV and 3.31/2.89 eV according to the difference of onsets between FL and PH spectra, respectively. The high enough singlet/triplet energies would promise efficient energy transfer from the host to guests through either the Förster or Dexter process in TADF-OLEDs. The properties of 2′-TRZSFX and 3′-TRZSFX were summarized in Table 1.

### 3.4. Carrier Transport Properties

**Figure 4:**
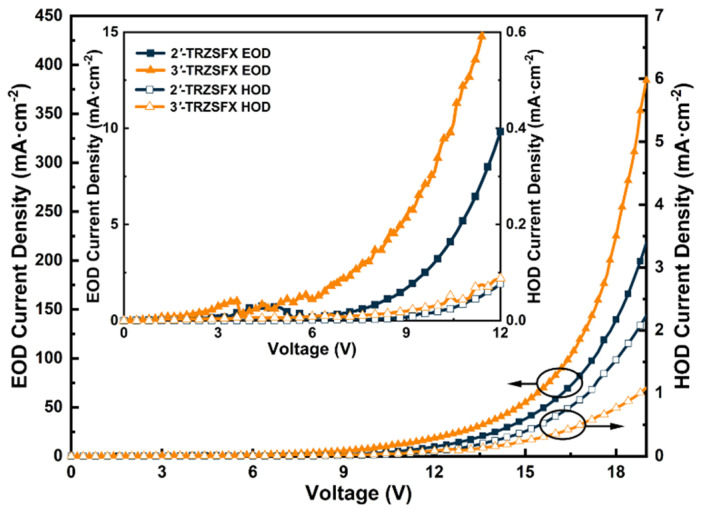
The curves of hole and electron current density (*J*) versus applied voltage (*V*) in single-carrier devices; (inset) *J*-*V* curves in the range of 0~12 V.

To evaluate the carrier transport properties of the host materials, we fabricated the hole-only devices (HODs) with the configuration of ITO/MoO_3_ (10 nm)/TAPC (50 nm)/*m*CP (10)/host (30 nm)/*m*CP (10 nm)/TAPC (50 nm)/MoO_3_ (10 nm)/Al (100 nm) and electron-only devices (EODs) with the configuration of ITO/LiF (1 nm)/TmPyPB (40 nm)/host (30 nm)/TmPyPB (40 nm)/LiF (1 nm)/Al (100 nm). Where TAPC was 1,1-*bis*(4-*bis*(4-methylphenyl)aminophenyl)cyclohexane, *m*CP was *N*,*N*′-dicarbazole-3,5-benzene and TmPyPB was 1,3,5-tri(m-pyridin-3-ylphenyl)benzene, respectively. The corresponding current density–voltage curves (*J-V*) are depicted in Figure 4. Overall observing the *J-V* curves, the 2′-TRZSFX and 3′-TRZSFX both showed remarkably high current density in EODs compared to the HODs, manifesting that the n-type design was successful via the introduction of the TRZ unit. Simultaneously, the current density of 3′-TRZSFX was higher than that of 2′-TRZSFX in EODs, and it was on the contrary in HODs. Further, we independently drew the *J-V* curves in the range of 0~12 V, as inserted in Figure 4. The current density of EOD of 3′-TRZSFX was still higher than that of 2′-TRZSFX; interestingly, the *J-V* curves of both hosts in HODs basically overlapped in this region. These results specified that the electron transport capacity of 3′-TRZSFX was superior to 2′-TRZSFX**,** on account of the coherent packing of TRZ units in solid state and the lower LUMO level of 3′-TRZSFX. Nevertheless, the balanced charge transport capacity of 2′-TRZSFX should not be overlooked. The electron and hole mobilities of 2′-TRZSFX and 3′-TRZSFX based on HODs, and EODs were calculated as fitting in Figure S5. The calculated mobilities were 4.038 × 10^−14^ and 6.165 × 10^−14^ cm^2^ V^−1^ s^−1^ for EODs, and 4.939 × 10^−15^ and 3.427 × 10^−15^ cm^2^ V^−1^ s^−1^ for HODs, for 2′-TRZSFX and 3′-TRZSFX, respectively.

### 3.5. OLED Characteristics

**Figure 5:**
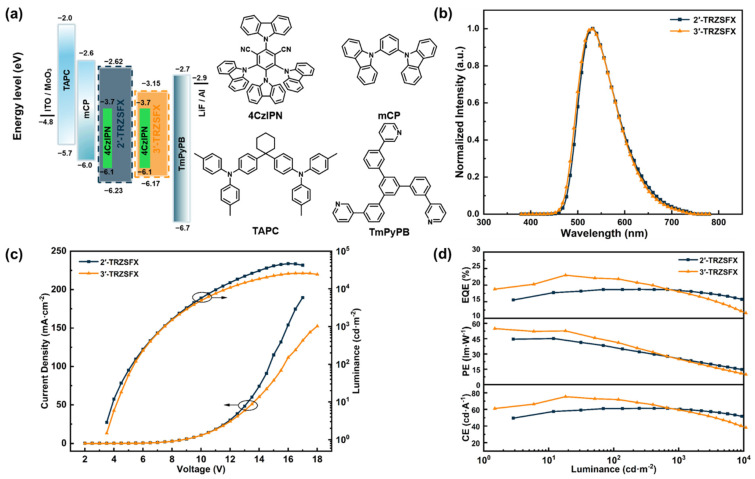
Device structures of green TADF OLED and performances. (**a**) Device architecture with HOMO and LUMO levels and the molecular structure of the materials. (**b**) EL spectrum of the devices at 10 V. (**c**) Current density–voltage–brightness. (**d**) EQE-, PE- and CE-luminance.

Encouraged by the high enough *T*_1_ energies and the n-type carrier transport characteristics of 2′-TRZSFX and 3′-TRZSFX, the TADF-OLED devices selecting 4CzIPN (1,2,3,5-tetrakis(carbazol-9-yl)-4,6-dicyanobenzene) as the emitter with the doping concentration of 15% were fabricated to evaluate the performance of the two hosts with the following structure: ITO/MoO_3_ (10 nm)/TAPC (40 nm)/*m*CP (10 nm)/host: 15 wt% 4CzIPN (30 nm)/TmPyPB (40 nm)/LiF (1 nm)/Al (100 nm), where 2′-TRZSFX and 3′-TRZSFX were used as the corresponding host materials for Devices A and B, respectively. In the devices, TAPC was used as the hole-transporting layer, *m*CP was used as the electron-blocking layer, and TmPyPB was used as the electron-injecting layer, respectively. Figure 5a schematically depicts the device architecture and the molecular structures of constituent materials.

Both devices exhibited almost the same green-emission peaking at 520 nm with stationary Commission International de l′Eclairage (CIE) coordinates at (0.35~0.36, 0.57~0.58) under the operating voltages of 2.0~16.0V, as shown in Figure 5b, Figures S5 and S6, demonstrating the effective energy transfer from the hosts to the 4CzIPN emitter. Figure 5c gives the current density–voltage–luminance (*J-V-L*) features of the TADF-OLEDs. Devices A and B possessed relatively low turn-on voltages of 3.4 and 3.5 V and the maximum luminance (*L*_max_) as high as 47,260 cd·m^−2^ and 25,556 cd·m^−2^, respectively. At the low operating-voltage region of 2.0~12.0 V, the two devices presented nearly similar current density and luminance; however, Device A showed higher current density and luminance with respect to Device B when the operating voltage exceeds 12.0 V.

Encouragingly, either Device A or Device B possessed impressive EL efficiencies, and the detailed EL performances were summarized in Table 2. As depicted in Figure 5d, Device B using 3′-TRZSFX to host the 4CzIPN emitter exhibited higher efficiencies relative to 2′-TRZSFX-based Device A in the luminance range of turn-on to ca. 1000 cd·m^−2^. Device B successfully achieved the peak efficiency with a maximum CE (*CE*_max_) of 75.5 cd·A^−1^, a maximum PE (*PE*_max_) of 55. 0 lm·W^−1^ and an *EQE*_max_ of 23.0%. Along with the increasing operating luminance, Device B still holds the CEs, PEs and EQEs as high as 72.3/59.7 cd·A^−1^, 42.1/24.7 lm·W^−1^ and 21.9/18.1% at the luminance of 100/1000 cd·m^−2^. The high enough efficiency and acceptable roll-off demonstrated that the *meta*-linkage better played the roles of n-type TRZ unit for electron transport and *T*_1_ maintaining. Interestingly, Device A based on the 2′-TRZSFX host showed a clear dominance in efficiency roll-off at the fully operating luminance with an *EQE*_max_ of 18.8% which was a little lower than Device B. According to the rigorous research by Cui and Adachi [18], 15 wt% 4CzIPN doping resulted in two orders of magnitude decrease in electron mobility and a thirtyfold increase in hole mobility compared with the neat film of n-type host SF3-TRZ, due to hole transport on the TADF molecules and electron transport via the host molecules. Combined with the characterization results of single-carrier devices of our hosts, we speculated that the same doping concentration likewise enhanced the hole transport in the EML layer of the two devices, especially for Device A in view of the better hole-transport capacity of 2′-TRZSFX relative to the 3′-TRZSFX under high operating voltage. The zigzag packing interaction between the donor (xanthene) and acceptor (TRZ) units of 2′-TRZSFX in the crystalline phase was beneficial to balancing the carrier transport in the EL layer, and then keeping a relatively lower roll-off under high operating voltages. Hence, the balanced carrier transport made the efficiency roll-off of Device A at 1000 cd·cm^−2^ as low as 2%. Furthermore, the luminance trend was positively associated with the current density trend [39], and then Device A had the maximum luminance of 47,260 cd·m^−2^.

In terms of relatively low LUMO level (−3.15 eV), planarized D-A structure and coherent packing mode, these would render 3′-TRZSFX strengthening n-type feature and facilitate electron transport and subsequent exciton combination in EML layer for Device B under low operating voltage. Of course, the feature also resulted in excessive electron transport and induced efficiency roll-off in high-bias regions (>12 V). Nonetheless, in consideration of the 18.1% EQE of Device B at 1000 cd·m^−2^, the 3′-TRZSFX would be a promising host material for high-efficiency TADF-OLED serving in flat-panel/flexible display.

## 4. Conclusions

In summary, a pair of SFX-based isomer-bearing TRZ units at its 2′- and 3′-positions were designed and synthesized as host materials for TADF-OLEDs. The positional effect of the TRZ unit on the molecular configuration/packing, intrinsic optoelectronic property and the characteristics of carrier transport were investigated in detail. It was found that the 3′-position substitution of TRZ, corresponding to 3′-TRZSFX, makes the HOMO level basically immobile and the LUMO level deepening relative to 2′-substituted isomer 2′-TRZSFX; meanwhile, the TRZ units presented continuous packing in the single crystal of 3′-TRZSFX. The respective features of relatively high electron transport capacity for 3′-TRZSFX and more balanced carrier transport for 2′-TRZSFX were revealed by single-carrier devices. The model TADF-OLEDs using the designed molecules to host 4CzIPN emitter both showed high *EQE*_max_s up to 18.8 and 23.0% for the 2′-TRZSFX- and 3′-TRZSFX-based devices, respectively, and the EL performance of the 3′-TRZSFX-based device was always higher than the 2′-TRZSFX-based device until the operating luminance of 1000 cd·m^−2^. When the operating luminance exceeded 1000 cd·m^−2^, the 2′-TRZSFX-based device presented higher efficiency and lower roll-off relative to the 3′-TRZSFX-based device. The revealed position effect of the TRZ unit on xanthene moiety provides a more informed strategy to develop SFX-based hosts for highly effective TADF-OLEDs.

## Figures and Tables

**Table 1:** Photophysical and electrochemical properties for the 2′-TRZSFX and 3′-TRZSFX.

Compound	*λ*_abs_*^a^* [nm]	*S*_1_/*T*_1_*^b^* [eV]	*E*_g_*^c^* [eV]	HOMO/LUMO *^d^* [eV]	*T*_d_ [°C]	*T*_m_ [°C]
2′-TRZSFX	270, 307, 324	3.44/2.82	3.68	−6.23/−2.62	375	298.8
3′-TRZSFX	272, 306, 335	3.31/2.89	3.61	−6.17/−3.15	370	295.5

*^a^* UV-vis absorbance peak measured in toluene solution (10^−5^ M). *^b^ S*_1_ energy determined from the onset of the fluorescence spectra at 298 K and the *T*_1_ determined from the onset of the phosphorescence spectra at 77 K. *^c^* Optical gap determined from the intersection point of the normalized absorption and fluorescence spectra. *^d^* The HOMO and LUMO levels were calculated according to the formula −[4.8 eV ± *E*_ox/red_ (vs. *E*_Fc/Fc+_)]; Fc/Fc^+^: ferrocene/ferrocenium.

**Table 2:** Summary of the device performance data of 2′-TRZSFX- and 3′-TRZSFX-based TADF OLEDs.

Device	*V ^a^* [V]	*CE ^b^* [cd·A^−1^]	*PE ^b^* [lm·W^−1^]	*EQE ^b^* [%]	*L*_max_ [cd·m^−2^]	*CIE ^c^*
Device A	3.4, 5.3, 7.5	61.5, 61.1, 60.3	45.4, 36.3, 25.2	18.8, 18.7, 18.4	47 260	(0.35, 0.57)
Device B	3.5, 5.4, 7.5	75.5, 72.3, 59.7	55.0, 42.1, 24.7	23.0, 21.9, 18.1	25 556	(0.36, 0.58)

*^a^* Operating voltage at luminance of onset, 100 cd·m^−2^, 1000 cd·m^−2^. *^b^* Values of CE, PE and EQE at their maximum, 100 cd·m^−2^, 1000 cd·m^−2^, 5000 cd·m^−2^. *^c^* Recorded at 10 mA·m^−2^.

## Data Availability

The data presented in this study are available on request from the corresponding author.

## Supplementary Materials

The following supporting information can be downloaded at: https://www.mdpi.com/article/10.3390/nano13182517/s1, Scheme S1: synthesis of 2′-TRZSFX and 3′-TRZSFX; Figure S1: various torsion angles in single crystals. (a) Xanthene planes dihedral angles of 2′-TRZSFX; (b) the planar fluorene intersection angles of 2′-TRZSFX; (c) xanthene planes dihedral angles of 3′-TRZSFX; (d) the planar fluorene intersection angles of 3′-TRZSFX. Figure S2: thermal gravity analysis of 2′-TRZSFX and 3′-TRZSFX; Figure S3: thermal gravity analysis of 2′-TRZSFX and 3′-TRZSFX; Figure S4: cyclic voltammetry of 2′-TRZSFX and 3′-TRZSFX; Figure S5: calculated electron and hole mobilities; Figure S6: corresponding emission colors on the CIE 1931 chromaticity diagram of 2′-TRZSFX and 3′-TRZSFX; Figure S7: CIE-brightness curve of two host devices; Table S1: single crystal data of 2′-TRZSFX; Table S2: single crystal data of 3′-TRZSFX; Figure S8: the ^1^H NMR spectrum of 2′-BrSFX (500 MHz, CDCl_3_); Figure S9: the ^1^H NMR spectrum of 3′-BrSFX (500 MHz, CDCl_3_); Figure S10: the ^1^H NMR spectrum of 2′-BpinSFX (500 MHz, CDCl_3_); Figure S11: the ^1^H NMR spectrum of 3′-BpinSFX (500 MHz, CDCl_3_); Figure S12: the ^1^H NMR spectrum of 2′-TRZSFX (500 MHz, CDCl_3_); Figure S13: the ^13^C NMR spectrum of 2′-TRZSFX(126 MHz, CDCl_3_); Figure S14: the ^1^H NMR spectrum of 3′-TRZSFX (500 MHz, CDCl_3_); Figure S15: the ^13^C NMR spectrum of 3′-TRZSFX (126 MHz, CDCl_3_); Figure S16: the HRMS spectrum of 3′-BrSFX; Figure S17: the HRMS spectrum of 2′-TRZSFX; Figure S18: the HRMS spectrum of 3′-TRZSFX.
