# Supplementary material for: Unraveling the Position Effect of Spiroxanthene-Based n-Type Hosts for High-Performance TADF–OLEDs
Source: Nanomaterials (Basel). 2023 Sep 8;13(18):2517. doi: 10.3390/nano13182517 (PMC10537283; doi:10.3390/nano13182517)
Supplement: Supplementary file 1 [file nanomaterials-13-02517-s001.zip › nanomaterials-2595068-supplementary.pdf]

Supplementary data

Unravelling the Position Effect of Spiroxanthene-Based n-Type Hosts for High-Performance TADF-OLEDs

Qing-Lin Liu[1,†], Yun Deng[2,†], Bao-Yi Ren[1,*], Xia Lan[1], Yue-Hong Zhang[1], Run-Da Guo[3,4,*], Chen-Sheng Li[1], Gang Xiong[1], Ya-Guang Sun[1] and Zujin Zhao[4]

1 College of Science, Key Laboratory of Inorganic Molecule-Based Chemistry of Liaoning Province, Shenyang University of Chemical Technology, Shenyang 110142, China. lql1347585318@163.com (Q.-L.L.); lan-xia0928@163.com (X.L.); zyhrrmhs@126.com (Y.-H.Z); lichensheng_hi@163.com (C.S.L.); xg@syuct.edu.cn (G.X.)

2 Frontiers Science Center for Flexible Electronics, Xi'an Institute of Flexible Electronics and Xi'an Institute of Biomedical Materials & Engineering, Northwestern Polytechnical University, Xi'an 710072, China. dengyun1981@163.com (Y.D.)

3 Wuhan National Laboratory for Optoelectronics, Huazhong University of Science and Technology, Wuhan 430074, China.

4 State Key Laboratory of Luminescent Materials and Devices, Key Laboratory of Luminescence from Molecular Aggregates of Guangdong Province, South China University of Technology, Guangzhou 510640, China. mszjzhao@scut.edu.cn (Z.Z)

† Qing-Lin Liu and Yun Deng contributed equally.

* Correspondence: renbaoyi@syuct.edu.cn (B.-Y.R); runda_guo@hust.edu.cn (R.-D.G);; Tel.: +86-024-8938-3302 (B.-Y.R)

1. Materials and methods

1.1 General Methods

The $^1H/^{13}C$ NMR spectra were obtained with a Bruker Avance500 II spectrometers at ambient temperature by utilizing deuterated chloroform ($CDCl_3$) as solvents and tetramethylsilane (TMS) as a standard. Mass spectra were obtained with a Waters Xevo G2-XS Tof. Single-crystal X-ray diffraction measurements of the crystals were performed with a Bruker Smart D8 Quest diffractometer equipped with a graphite monochromator. The determination of the unit cell parameters and the data collection were performed with Mo K_α radiation (λ = 0.71073 Å). UV-vis absorption spectra were measured using a UV-2550 Spectrophotometer. PL spectra were performed using a LS55 Spectrofluorophotometer. Cyclic voltammetry (CV) experiments were employed to evaluate the energy levels with a CH1760E Microcomputer based Electrochemical Analyzer, using *n*-Bu_4NPF_6 (0.1 M) in dry dichloromethane as electrolyte solution and ferrocene as an internal reference. The experiments were carried out in a conventional three-electrode configuration, using a glass carbon disk as the working electrode, a saturated calomel electrode (SCE) as the reference electrode and a Pt wire as the counter electrode at a scan rate of 100 m Vs^{-1} under the protection of nitrogen. The **2′-TRZSFX** and **3′-TRZSFX** were dissolved in dichloromethane on the working electrode for measurements. The energy levels of the compounds were estimated by the equation: (assumed the energy level of $FeCp_2^{+/0}$ to be 4.8 eV in vacuum): $E_{HOMO} = -[E_{onset}^{ox} + 4.8]$ (eV); $E_{LUMO} = -[E_{onset}^{red} + 4.8]$ (eV). Thermogravimetric analyses (TGA) were determined using a METTL TOLEDO thermogravimetric Analyzer under a heating rate of 10 °C min^{-1} and a nitrogen flow rate of 50 cm^3 min^{-1}.

1.2 OLED fabrication

All purchased reagents and raw materials used in the synthesis process were used without further purification unless other stated. All reaction processes were carried out under a protection of nitrogen atmosphere. The final compounds were further synthesized through Suzuki cross-coupling reaction, as shown in the experimental section. The final compounds with little impurity were preliminarily purified by means of silica gel column chromatography, then repeatedly using recrystallization, finally sublimation by gradient heating under vacuum condition. For devices fabrication, the used ITO coated glass substrates (20 Ω $square^{-1}$), MoO_3, LiF, TAPC, TmPyPB and *m*CP were commercially available. The ITO substrates were dipped into acetone, isopropyl alcohol and deionized water orderly, and clean with ultrasonic washer for 30 minutes. Before preparing devices, the ITO substrates were pretreated with oxygen plasma for 5 minutes. The devices were fabricated by evaporating organic materials, which deposited in the vacuum of 2×10^{-4} Pa and monitoring *via* crystal oscillator system. The *J-V-L* property and EL spectra of the devices were measured by a Keithley 2400 source meter equipped with a PR655 spectrometer, and the emitting areas were 0.09 cm^2 which determined by the overlaps of two electrodes. All measurements were carried out at room temperature under ambient conditions.

1.3 Synthesis

All solvents and chemicals, unless otherwise indicated, were purchased from commercial suppliers without further purification. All reactions were monitored by thin layer chromatographic analysis by thin-layer chromatography, which was visualized by a UV lamp at 254 or 365 nm. Flash column chromatography was performed on glass column of silica gel (200 ~ 300 mesh) and solvent ratios

were expressed in volume to volume.

Synthesis of SFX *(spiro[fluorene-9,9'-xanthene)*: phenol (23.11 g, 245.55 mmol) , 9*H*-fluoren-9-one (7.50 g, 41.62 mmol) and methanesulfonic acid (10.64 ml, 163.98 mmol) were mixed and heated at 150 °C in Ar for 12 h. Then, the reaction was quenched through poured into ethanol at a high temperature and the mixture was then stirred for 10 h. Subsequently, the filtrate obtained after filtration is dried by $MgSO_4$ and concentration-process by rotary evaporation. In the last, performed a purification procedure that is silica gel column chromatography using an eluent of petroleum ether–dichloromethane mixture (4: 1, v/v), and obtained a white solid SFX (7.3 g, 52.8 %). ^{1}H NMR (500 MHz, $CDCl_3$) δ 7.81 (d, J = 7.6 Hz, 2H), 7.38 (td, J = 7.4, 1.4 Hz, 2H), 7.27 – 7.15 (m, 8H), 6.78 (ddd, J = 8.2, 6.9, 1.5 Hz, 2H), 6.42 (dd, J = 7.9, 1.6 Hz, 2H).

Synthesis of 3′-BrSFX *(3′-bibromospiro[fluorene-9,9′-xanthene)*: phenol (5.22 g, 55.49 mmol), 3-bromophenol (12 g, 69.37 mmol), 9*H*-fluoren-9-one (2.50 g, 13.87 mmol) and methanesulfonic acid (6.67 g, 69.37 mmol) were mixed and heated at 150 °C in Ar for 12 h. Then, the reaction was quenched through poured into ethanol at a high temperature and the mixture was then stirred for 10 h. Subsequently, the filtrate obtained after filtration is dried by $MgSO_4$ and concentration-process by rotary evaporation. In the last, performed a purification procedure that is silica gel column chromatography using an eluent of petroleum ether–dichloromethane mixture (1:3, v/v), and obtained a white solid 3′-BrSFX (0.86 g, 15 %). ^{1}H NMR (500 MHz, $CDCl_3$) δ 7.80 (d, J = 7.6 Hz, 2H), 7.44 – 7.34 (m, 3H), 7.24 – 7.17 (m, 4H), 7.14 (d, J = 7.6 Hz, 2H), 6.88 (dd, J = 8.5, 2.0 Hz, 1H), 6.79 (ddd, J = 8.2, 6.1, 2.3 Hz, 1H), 6.39 (d, J = 7.8 Hz, 1H), 6.26 (d, J = 8.3 Hz, 1H). HRMS for $C_{25}H_{15}BrO$: $[M + Na]^+$ calcd. 433.0199, found 433.0208.

Synthesis of 2′-BrSFX *(2′-bibromospiro[fluorene-9,9′-xanthene)*: SFX (5 g, 15.04 mmol), $NaBrO_3$ (2.81 g, 16.85 mmol) and NaBr (3.1 g, 30.08 mmol) in chloroform were mixed and in Ar for 1 h. Then, extracted by CH_2Cl_2. Subsequently, concentration-process by rotary evaporation. In the last, performed a purification procedure that is silica gel column chromatography using an eluent of petroleum ether-dichloromethane mixture (4 :1, v/v), and obtained a white solid 2′-BrSFX (4 g, 64.9%). ^{1}H NMR (500 MHz, $CDCl_3$) δ: 7.83 (dd, J = 7.9, 4.2 Hz, 3H), 7.43 (dt, J = 14.6, 7.5 Hz, 3H), 7.30 (dd, J = 8.8, 2.2 Hz, 2H), 7.25 – 7.11 (m, 7H), 6.80 (dd, J = 8.0, 6.0 Hz, 1H), 6.50 (dt, J = 15.0, 2.1 Hz, 2H), 6.40 (d, J = 7.9 Hz, 1H).

Synthesis of 2′-BpinSFX *(4,4,5,5-tetramethyl-2-(spiro[fluorene-9,9′-xanthen]-2′-yl)-1,3,2-dioxaborolane)*: 2′-BrSFX (2.50 g, 6.08 mmol), bis(pinacolato)diboron (3.09 g, 12.16 mmol), 1,1′-bis(diphenylphosphinoferrocene)dichloro-palladium (II) (337 mg 0.61 mmol), palladium acetate (68.23 mg, 303.92 μmol) and potassium acetate (1.43 mg, 14.59 mmol)) in 1,4-dioxane (70 ml) were mixed and heated at 90 °C in Ar for 12 h. Then, cooled the temperature to 25°C and extracted by CH_2Cl_2. Subsequently, made drying-treatment with the obtained organic extract by $MgSO_4$ and concentration-process by rotary evaporation. In the last, performed a purification procedure that is silica gel column chromatography using an eluent of petroleum ether–dichloromethane mixture (4:3, v/v), and obtained a white solid 2′-BpinSFX (1.75 g, 62.8 %). ^{1}H NMR (500 MHz, $CDCl_3$) *δ*: 7.83 (d, J = 7.6 Hz, 2H), 7.69 (dd, J = 8.2, 1.5 Hz, 1H), 7.39 (td, J = 7.4, 1.2 Hz, 2H), 7.27 – 7.13 (m, 7H), 6.89 (d, J = 1.6 Hz, 1H), 6.76 (td, J = 7.5, 7.1, 1.5 Hz, 1H), 6.34 (dd, J = 7.9, 1.5 Hz, 1H), 1.21 (s, 12H).

Synthesis of 3′-BpinSFX*(4,4,5,5-tetramethyl-2-(spiro[fluorene-9,9′-xanthen]-3′-yl)-1,3,2-dioxaborolane)*: 3′-BrSFX (0.77 g, 1.87 mmol), bis(pinacolato)diboron (0.95 g, 3.73 mmol), 1,1'-bis(diphenylphosphinoferrocene)dichloro-palladium (II) (0.05 g, 0.06 mmol), and potassium acetate

(0.84 g, 8.58 mmol) in 1,4-dioxane (55 ml) were mixed and heated at 90 °C in Ar for 21 h. Then, cooled the temperature to 25°C and extracted by CH_2Cl_2. Subsequently, made drying-treatment with the obtained organic extract by $MgSO_4$ and concentration-process by rotary evaporation. In the last, performed a purification procedure that is silica gel column chromatography using an eluent of petroleum ether–dichloromethane mixture (4:3, v/v), and obtained a white solid 3′-BpinSFX (0.49 g, 58 %). ^{1}H NMR (500 MHz, $CDCl_3$) δ 7.80 (d, J = 7.6 Hz, 2H), 7.69 (d, J = 1.2 Hz, 1H), 7.37 (td, J = 7.4, 1.3 Hz, 2H), 7.22 – 7.13 (m, 7H), 6.76 (ddd, J = 8.2, 6.7, 1.8 Hz, 1H), 6.43 – 6.36 (m, 2H), 1.32 (s, 13H).

Synthesis of 2′-TRZSFX *(2,4-diphenyl-6-(spiro[fluorene-9,9'-xanthen]-2'-yl)-1,3,5-triazine)*: 2-chloro-4,6-diphenyl-1,3,5-triazine (0.18 g, 0.66 mmol), 2′-BpinSFX (0.32 g, 0.70 mmol), tetrakis(triphenylphosphine)palladium (0) (0.04 g, 0.04 mmol) and potassium carbonate (0.23 g, 1.66 mmol) in a mixed solvent (H_2O: THF = 1:3) (6 ml) were mixed and heated at 80 °C in Ar for 3 h. Then, cooled the temperature to 25°C and extracted by CH_2Cl_2. Subsequently, made drying-treatment with the obtained organic extract by $MgSO_4$ and concentration-process by rotary evaporation. In the last, performed a recrystallization procedure in a mixed solvent of dichloromethane / cyclohexane, and obtained a yellow solid 2′-TRZSFX in 62% yield (0.18 g). ^{1}H NMR (500 MHz, $CDCl_3$) δ 8.56 (ddd, J = 22.9, 8.6, 1.9 Hz, 5H), 7.92 – 7.84 (m, 3H), 7.60 – 7.38 (m, 9H), 7.31 (dd, J = 8.2, 1.4 Hz, 1H), 7.27 (d, J = 4.0 Hz, 5H), 6.87 (td, J = 7.6, 1.4 Hz, 1H), 6.58 (dd, J = 7.8, 1.6 Hz, 1H), 1.55 (s, 2H). ^{13}C NMR (126 MHz, $CDCl_3$) δ 171.21, 170.57, 155.00, 154.44, 151.43, 139.88, 136.14, 132.37, 131.43, 129.47, 128.90, 128.82, 128.53, 128.38, 128.01, 127.87, 126.00, 125.69, 125.20, 123.95, 120.15, 117.17, 116.98, 77.31, 77.05, 76.80, 54.49, 29.76, 1.07. HRMS (MALDI-TOF) for $C_{40}H_{25}N_3O$: $[M + H]^+$ calcd. 564.2071, found 564.2077.

Synthesis of 3′-TRZSFX *(2,4-diphenyl-6-(spiro[fluorene-9,9'-xanthen]-3'-yl)-1,3,5-triazine)*: 2-chloro-4,6-diphenyl-1,3,5-triazine (0.43 g, 1.62 mmol), 3′-BpinSFX (0.49 g, 1.08 mmol), tetrakis(triphenylphosphine)palladium (0) (0.25 g, 0.22 mmol), potassium fluoride dihydrate (0.46 g, 4.94 mmol) and potassium carbonate (0.68 g, 4.94 mmol) in a mixed solvent (H_2O: THF: toluene = 1: 3: 3) (35 ml) were mixed and heated at 90 °C in Ar for 18 h. Then, cooled the temperature to 25°C and extracted by CH_2Cl_2. Subsequently, made drying-treatment with the obtained organic extract by $MgSO_4$ and concentration-process by rotary evaporation. In the last, performed a recrystallization procedure in a mixed solvent of dichloromethane / cyclohexane, and obtained a yellow solid 3′-TRZSFX in 80% yield (0.49 g). ^{1}H NMR (500 MHz, $CDCl_3$) δ 8.80 – 8.70 (m, 4H), 8.63 (d, J = 1.7 Hz, 1H), 8.16 (dd, J = 8.2, 1.8 Hz, 1H), 7.85 (d, J = 7.7 Hz, 2H), 7.66 – 7.53 (m, 6H), 7.41 (ddd, J = 7.9, 6.4, 2.0 Hz, 2H), 7.32 (dd, J = 8.3, 1.3 Hz, 1H), 7.26 – 7.14 (m, 5H), 6.83 (s, 1H), 6.61 (d, J = 8.2 Hz, 1H), 6.47 (dd, J = 7.9, 1.6 Hz, 1H), 1.25 (s, 2H). ^{13}C NMR (126 MHz, $CDCl_3$) δ 171.74, 171.11, 154.89, 151.81, 151.51, 139.86, 136.69, 136.24, 132.69, 129.69, 129.09, 128.77, 128.65, 128.51, 128.48, 128.19, 128.15, 125.89, 124.62, 123.79, 123.69, 120.22, 117.51, 116.98, 77.41, 77.16, 76.91, 54.52, 29.85, 29.52, 14.29, 1.18. HRMS (MALDI-TOF) for $C_{40}H_{25}N_3O$: $[M + H]^+$ calcd. 564.2071, found 564.2072.

2. Supporting figures

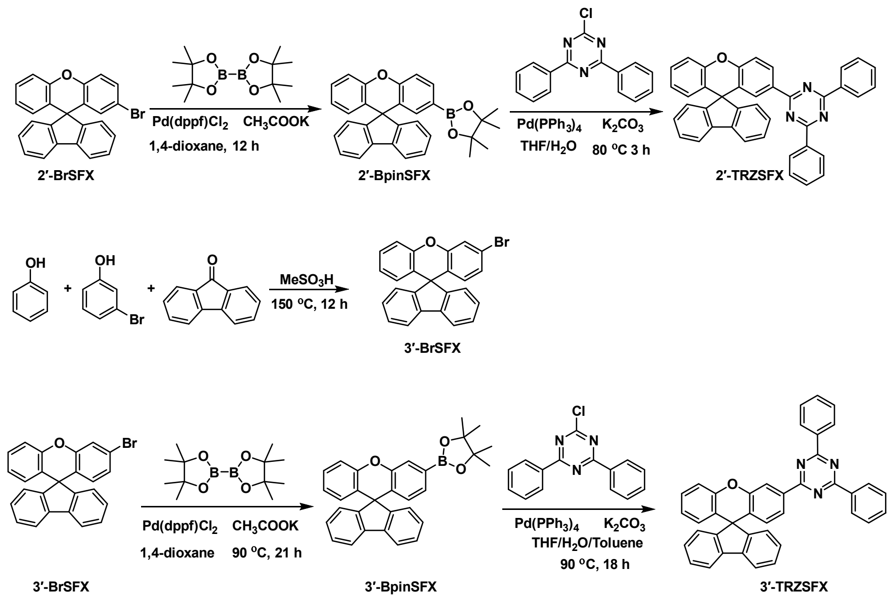

Scheme S1 Synthesis of **2′-TRZSFX** and **3′-TRZSFX**.

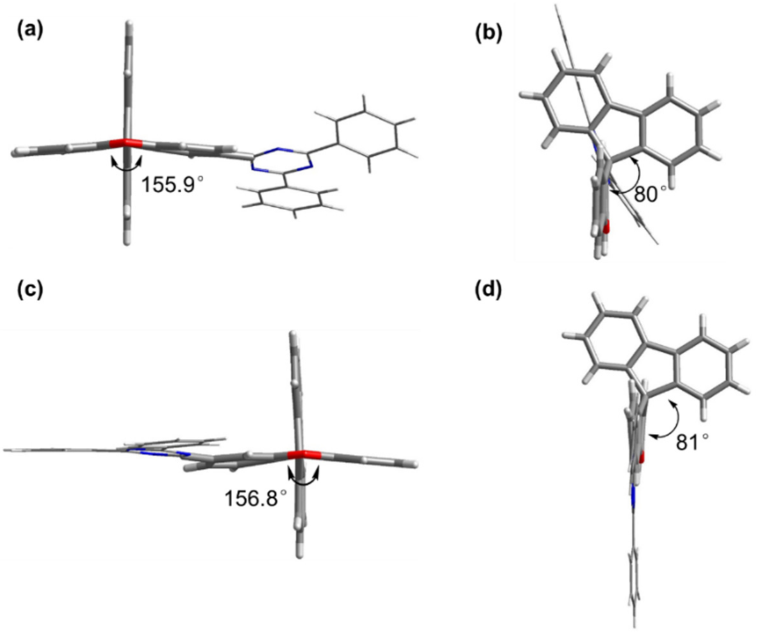

Figure S1 Various torsion angles in single crystals **(a)** Xanthene planes dihedral angles of **2′-TRZSFX**; **(b)** The planar fluorene intersection angles of **2′-TRZSFX**; **(c)** Xanthene planes dihedral angles of **3′-TRZSFX**; **(d)** The planar fluorene intersection angles of **3′-TRZSFX**.

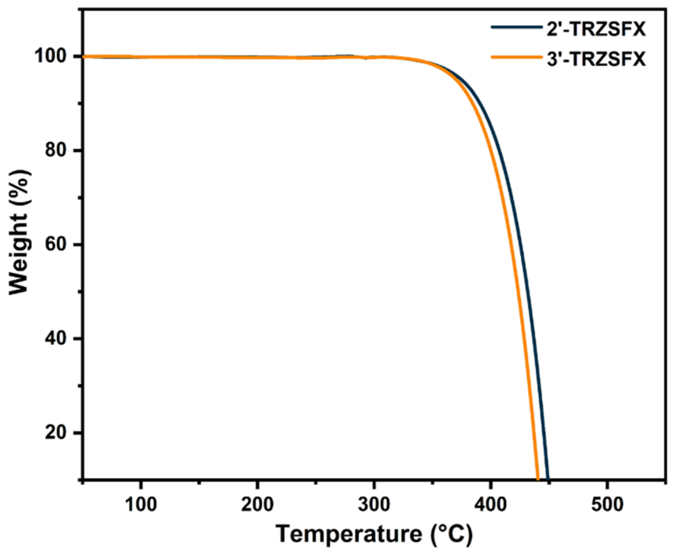

Figure S2 Thermal gravity analysis of **2′-TRZSFX** and **3′-TRZSFX**.

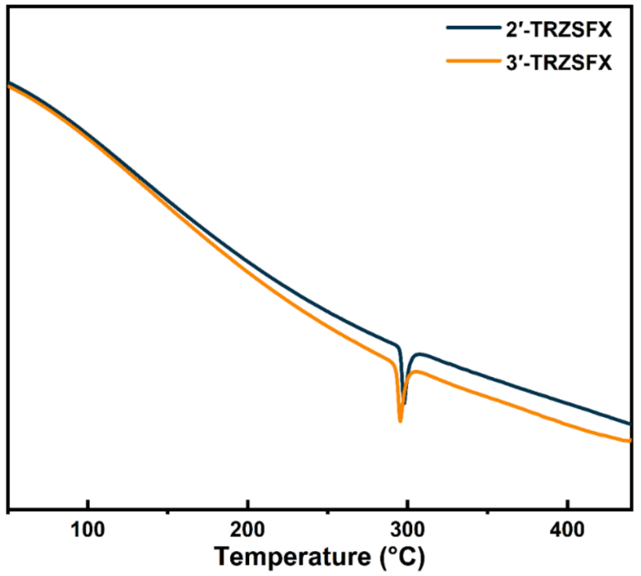

Figure S3 Thermal gravity analysis of **2′-TRZSFX** and **3′-TRZSFX**.

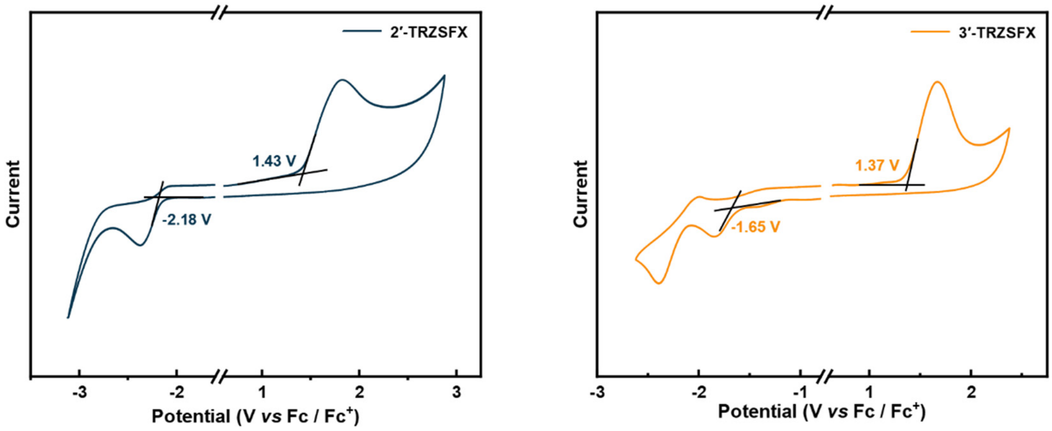

Figure S4 Cyclic voltammetry of **2′-TRZSFX** and **3′-TRZSFX**.

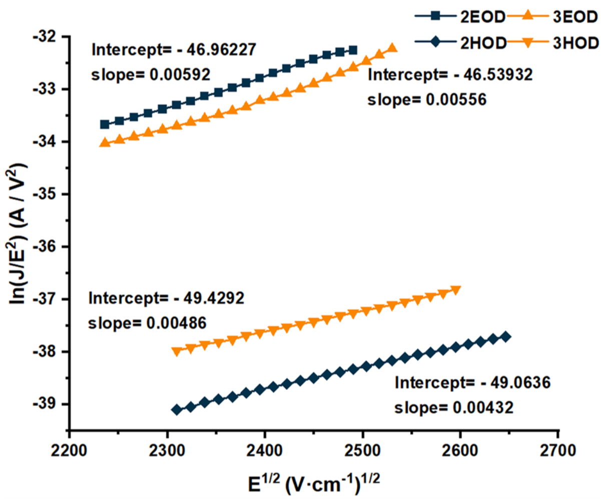

	μ_{0e}	β_e	μ_{0h}	β_h
2′-TRZSFX	4.038×10^{-14}	0.00592	4.939×10^{-15}	0.00432
3′-TRZSFX	6.165×10^{-14}	0.00556	3.427×10^{-15}	0.00486

Figure S5 Calculated electron and hole mobilities.

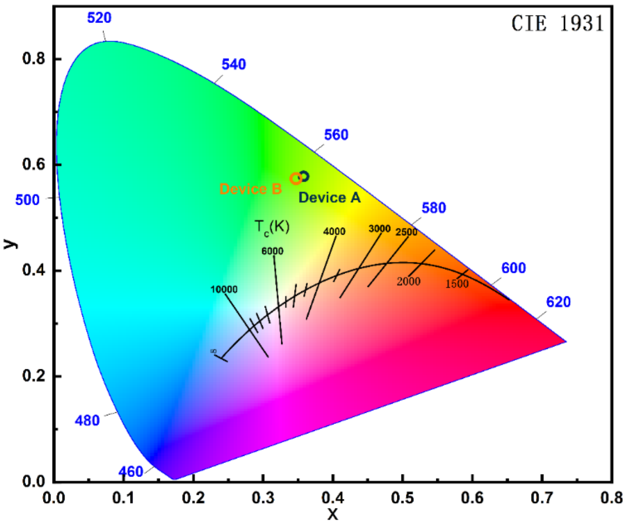

Figure S6 Corresponding emission colors on the CIE 1931 chromaticity diagram of **2′-TRZSFX** and **3′-TRZSFX**.

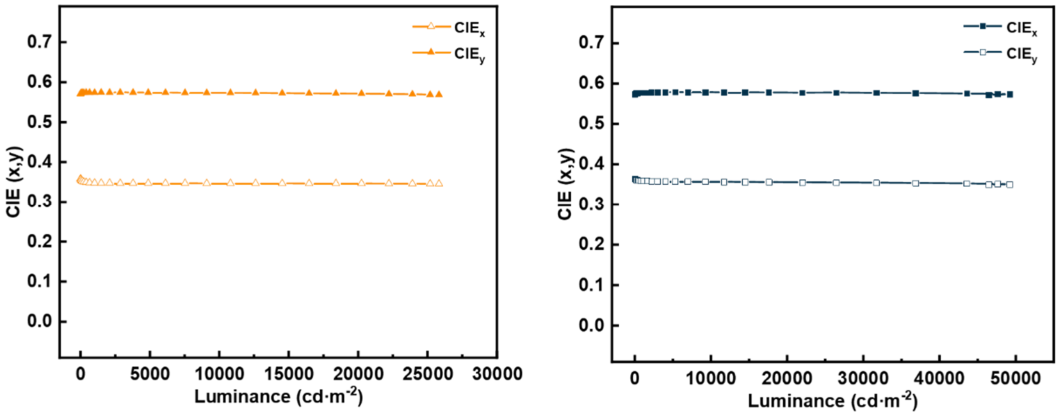

Figure S7 CIE-brightness curve of two host devices.

Table S1 Single crystal data of **2′-TRZSFX**

**Compound**	**CCDC 2196212**
Formula moiety	$C_{40}H_{25}N_3O$
Formula weight	1076.93
Crystal system	triclinic
Space group	P1
a/Å	9.4271(16)
b/Å	10.6008(18)
c/Å	15.149(2)
α/°	96.562(7)
β/°	105.618(6)
γ/°	91.168(7)
Volume/Å^3	1446.4(4)
Z	2
Crystal density	1.294
F(000)	588
Crystal size (mm)	0.2

Table S2 Single crystal data of **3′-TRZSFX**

**Compound**	**CCDC: 2196215**
Formula moiety	$C_{40}H_{25}N_3O$
Formula weight	2254.51
Crystal system	triclinic
Space group	P1
a/Å	10.5737(9)
b/Å	14.5991(12)
c/Å	20.0101(17)
α/°	79.010(4)
β/°	83.425(4)
γ/°	73.052(3)
Volume/Å^3	2894.8(4)
Z	4
Crystal density	1.293
F(000)	1176
Crystal size (mm)	0.2

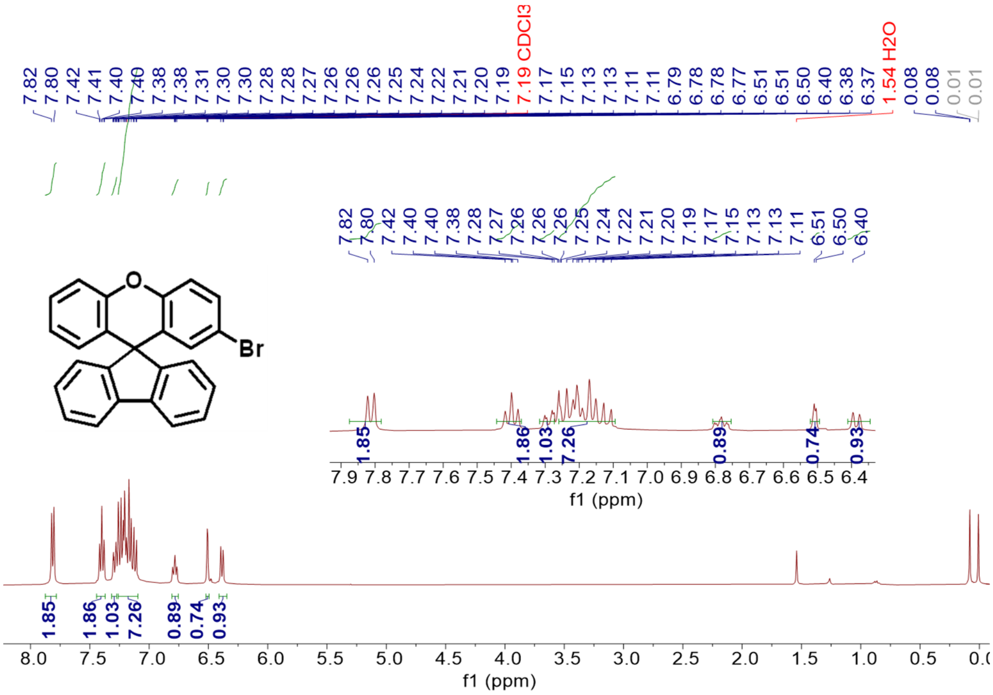

Figure S8 The ^{1}H NMR spectrum of **2′-BrSFX** (500 MHz, $CDCl_3$).

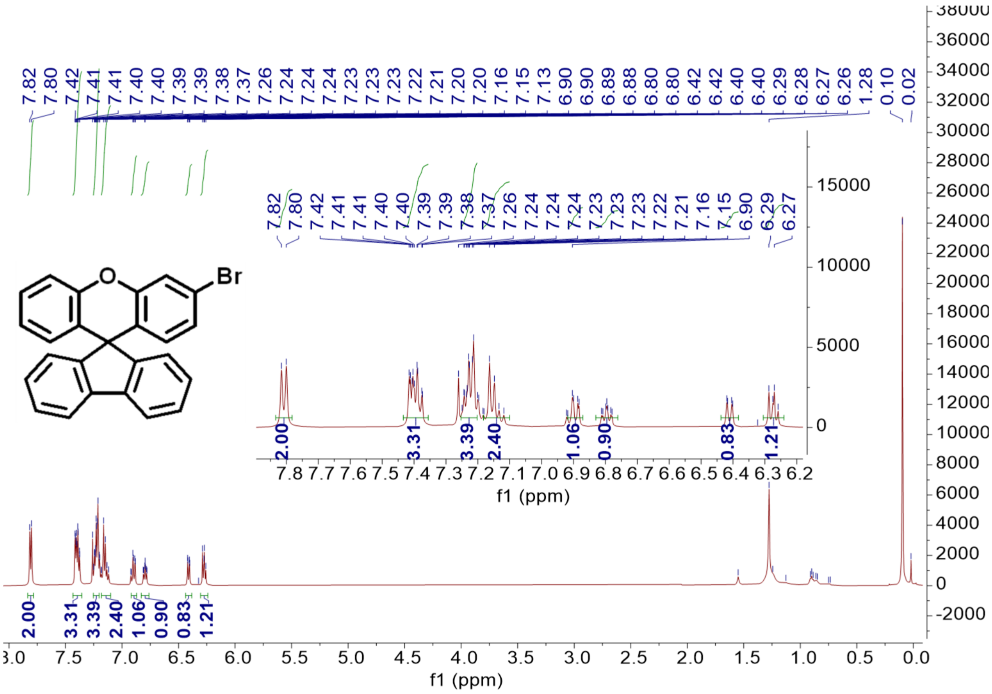

Figure S9 The ^{1}H NMR spectrum of **3′-BrSFX** (500 MHz, $CDCl_3$).

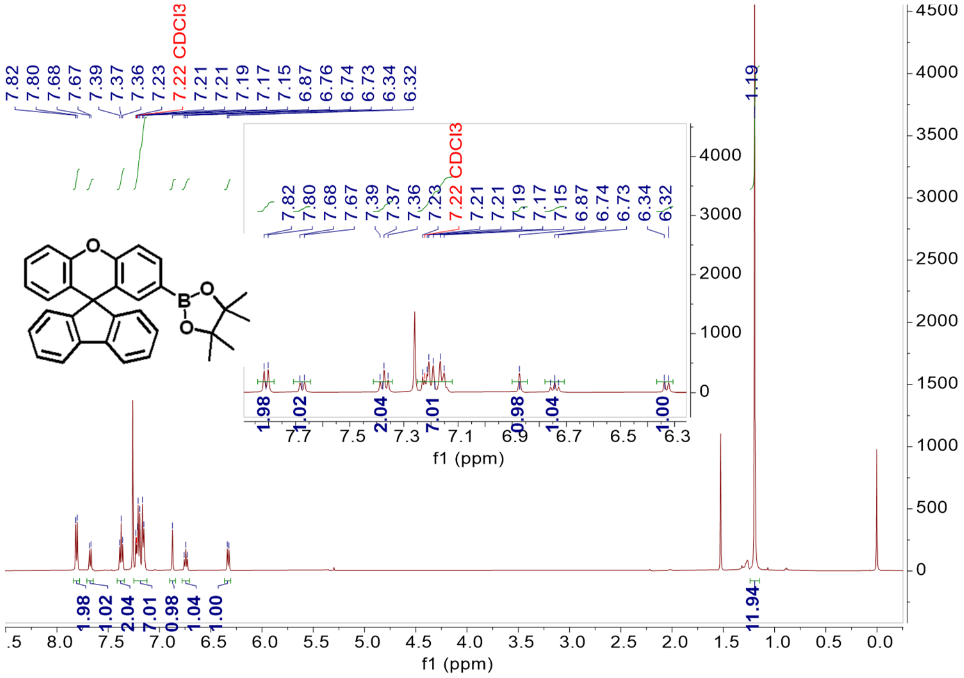

Figure S10 The ^{1}H NMR spectrum of **2′-BpinSFX** (500 MHz, $CDCl_3$).

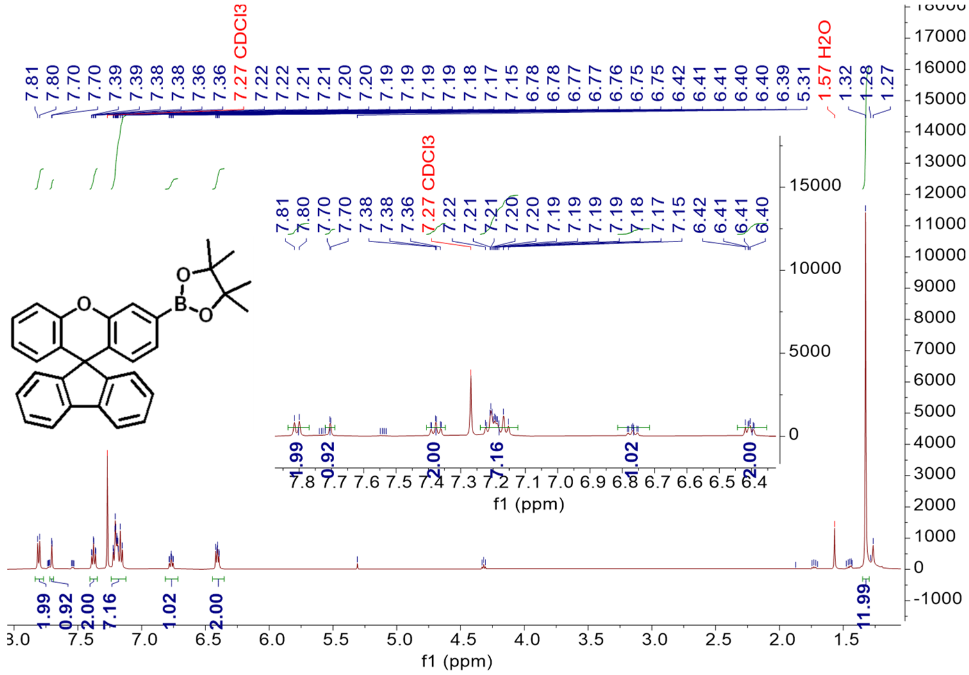

Figure S11 The ^{1}H NMR spectrum of **3′-BpinSFX** (500 MHz, $CDCl_3$).

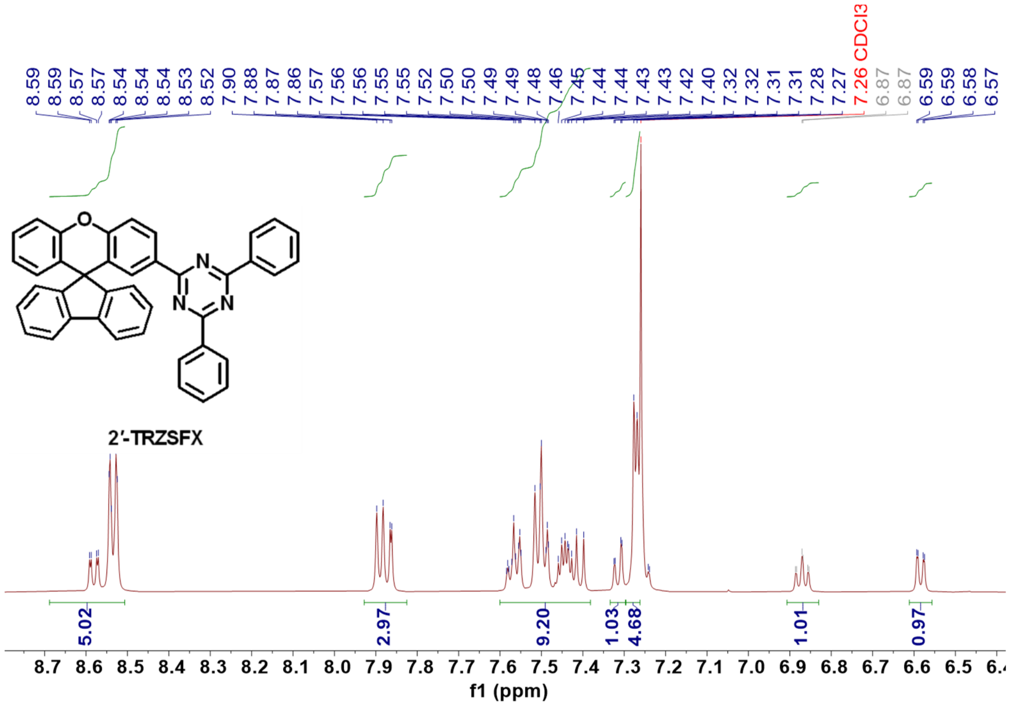

Figure 12 The ^{1}H NMR spectrum of **2′-TRZSFX** (500 MHz, $CDCl_3$).

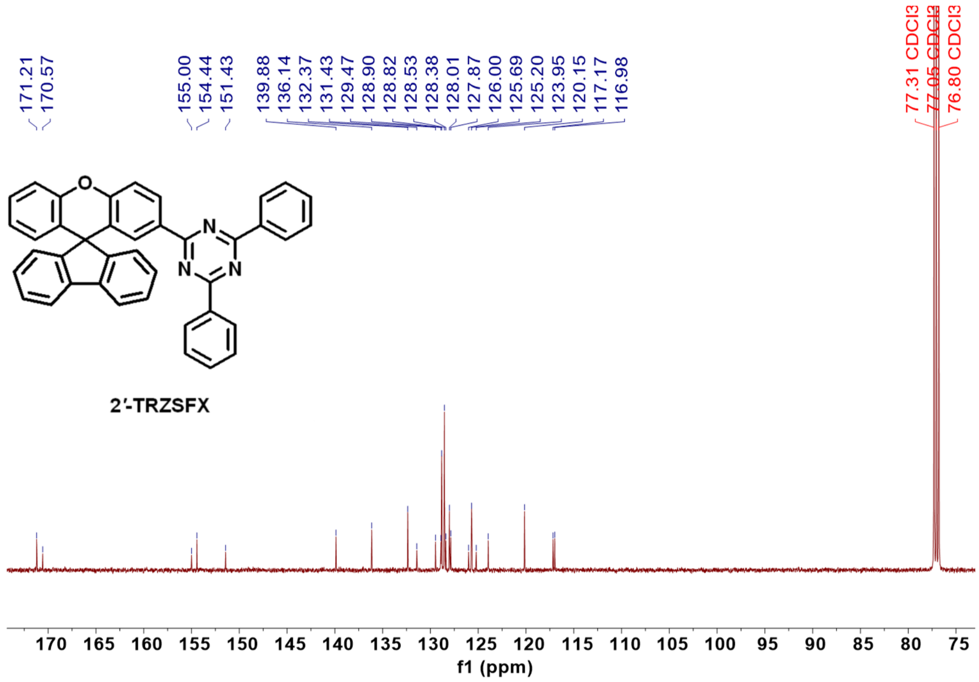

Figure S13 The ^{13}C NMR spectrum of **2′-TRZSFX**(126 MHz, $CDCl_3$).

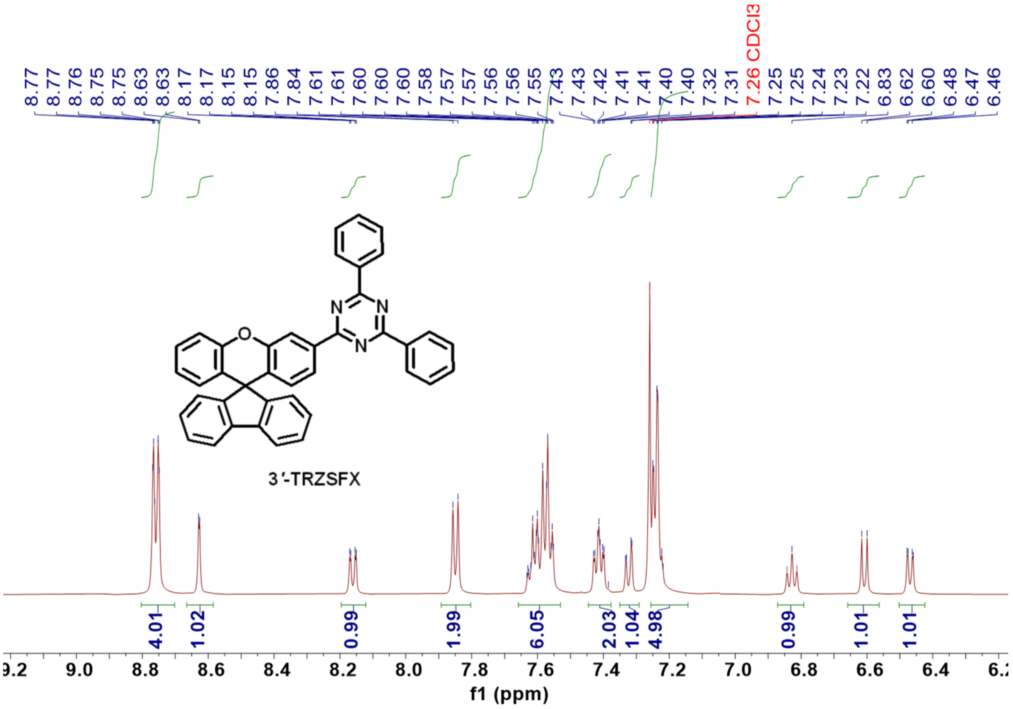

Figure S14 The ^{1}H NMR spectrum of **3′-TRZSFX** (500 MHz, $CDCl_3$).

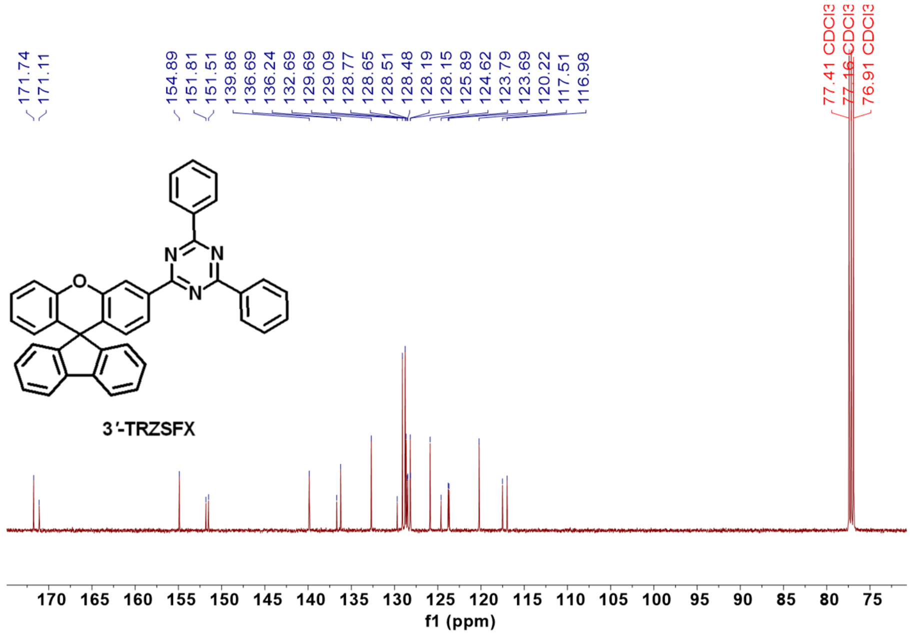

Figure S15 The ^{13}C NMR spectrum of **3′-TRZSFX** (126 MHz, $CDCl_3$).

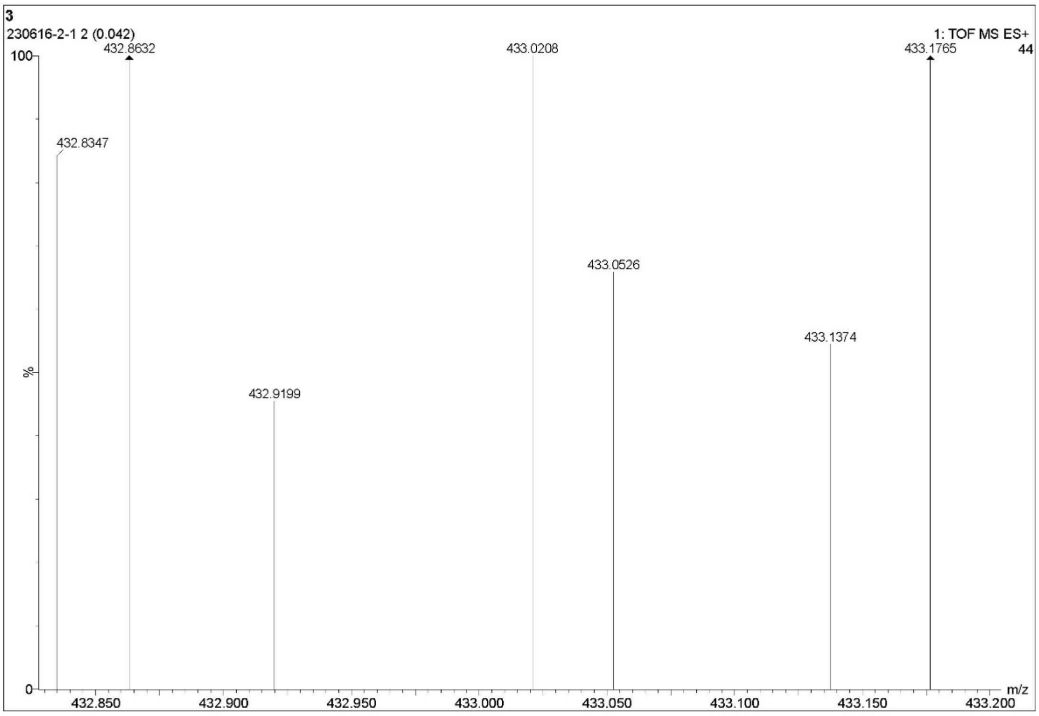

Figure S16 The HRMS spectrum of **3′-BrSFX**.

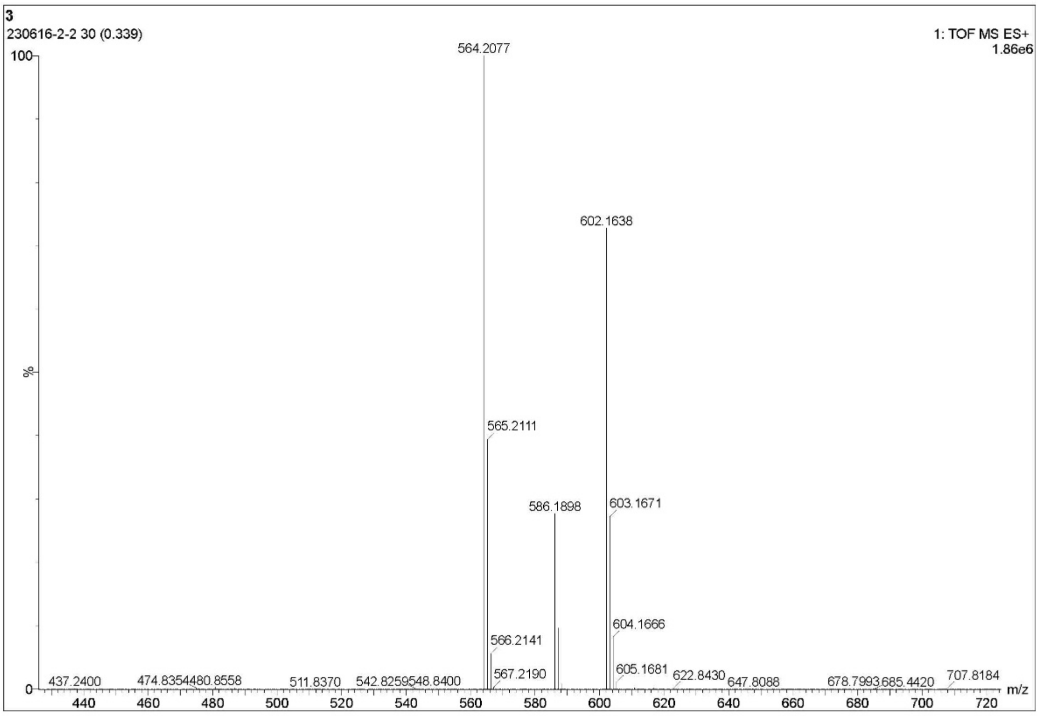

Figure S17 The HRMS spectrum of **2′-TRZSFX**.

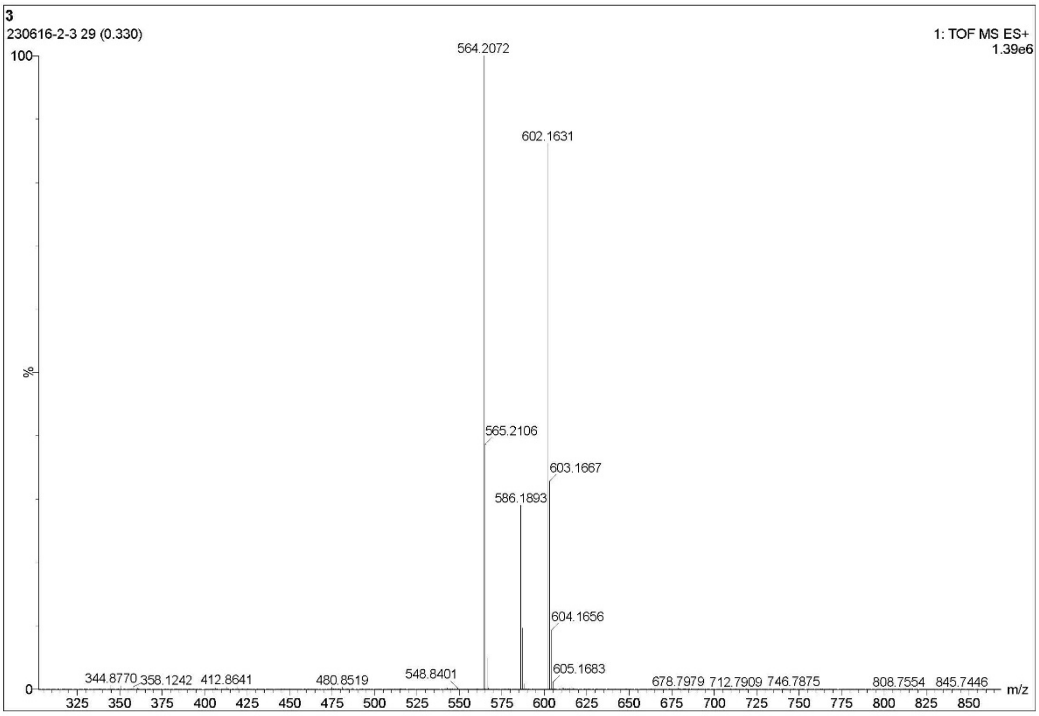

Figure S18 The HRMS spectrum of **3′-TRZSFX**.
